# NSAID Tolerance Can Be Restored in NECD Even in the Absence of Complete Remission of Urticaria

**DOI:** 10.1002/clt2.70216

**Published:** 2026-10-21

**Authors:** Merve Hormet Igde, Pelin Korkmaz, Semra Demir, Osman Ozan Yegit, Derya Ünal, Merve Dilşad Atasever, Ayşe Feyza Aslan, Leyla Bölek, Selin Ece Güzel, Nevzat Kahveci, Mehmet Emin Sezgin, Şule Çelik Kamacı, Esra Kaya, Bircan Erden, Asli Gelincik

**Affiliations:** ^1^ Department of Internal Medicine Division of Allergy and Clinical Immunology Istanbul University Istanbul Faculty of Medicine Istanbul Türkiye

## Abstract

**Background/Aim:**

Data on the course of nonsteroidal anti‐inflammatory drug (NSAID) hypersensitivity in NSAID‐exacerbated cutaneous disease (NECD) are limited. We aimed to evaluate NSAID tolerance in NECD patients with well‐controlled urticaria.

**Methods:**

142 patients with well‐controlled urticaria [Urticaria Control Test (UCT) ≥ 12; Urticaria Activity Score over 7 days (UAS7) ≤ 6] underwent a single blind, placebo‐controlled acetylsalicylic acid (ASA) provocation test. Patients were categorized into three groups based on their treatment; omalizumab, antihistamine, or no treatment.

**Results:**

The median age of the patients was 40 years (IQR 31.5–52), and 79.6% were female. The median disease duration of chronic spontaneous urticaria (CSU) was 4 years (IQR 2–8). The ASA provocation test was positive in 29 patients (20.4%). Positive patients had lower UCT and higher UAS7 scores (both *p* < 0.001). ASA provocation test positivity was more common in the antihistamine group compared to both the omalizumab (*p* = 0.001) and untreated groups (*p* < 0.001). ROC analysis showed that UCT ≤ 15 predicted ASA provocation test positivity with 72.4% sensitivity and 83.2% specificity, while UAS7 ≥ 4 predicted positivity with 72.4% sensitivity and 86.7% specificity. The negative predictive values (NPV) were 92.2% and 92.5%, respectively.

**Conclusion:**

To our knowledge, this is the first study to demonstrate, in a large NECD cohort, that NSAID tolerance can be achieved in patients with well‐controlled urticaria under different treatment modalities, regardless of complete remission status. UCT ≤ 15 and UAS7 ≥ 4 are useful clinical predictors of provocation test positivity and, given their high NPVs, are particularly valuable for excluding positivity.

## 1 Introduction

Hypersensitivity to acetylsalicylic acid (ASA) and other nonsteroidal anti‐inflammatory drugs (NSAIDs) affects approximately 1%–3% of the general population, whereas 20%–30% of patients with chronic spontaneous urticaria (CSU) experience urticarial flares following exposure [1, 2]. In the European Academy of Allergy and Clinical Immunology/European Network on Drug Allergy (EAACI/ENDA) classification, NSAID‐exacerbated cutaneous disease (NECD) represents a cross‐reactive CSU phenotype in which reactions are typically triggered by strong cyclooxygenase‐1 (COX‐1) inhibitors across different chemical classes. This is thought to occur via COX‐1 blockade, leading to a shift in arachidonic acid metabolism toward cysteinyl leukotrienes [1, 3]. Although weak COX‐1 inhibitors and selective COX‐2 inhibitors are tolerated in many patients, these agents are not universally effective, and intolerance may still occur in individuals with multiple NSAID reactions [4]. Current guidelines recommend avoidance of known triggers, including NSAIDs; however, the clinical necessity of these agents for pain and inflammation management makes such an approach challenging [5].

One of the key challenges in understanding the natural course of CSU and NSAID tolerance is the remission definition proposed by the World Allergy Organization (WAO), which requires a minimum of 6 months without symptoms and without the need for treatment [6]. In clinical practice, however, achieving remission is often prolonged and unpredictable, leading to unnecessary NSAID avoidance in many patients with NECD. Although current guidelines do not support this approach, observational evidence suggests that, in certain clinical contexts, disease control (e.g., Urticaria Control Test [UCT] ≥ 12, Urticaria Activity Score over 7 days [UAS7] ≤ 6) may influence the appropriateness of provocation testing [5, 7].

Recent evidence suggests that NSAID reactivity fluctuates dynamically in parallel with the underlying activity of CSU [7, 8]. Furthermore, the treatment of CSU has been associated with improved outcomes in NSAID‐induced reactions. Clinical control achieved with H1‐antihistamines has been shown to reduce NSAID‐induced exacerbations, and some patients can regain tolerance to previously culprit NSAIDs [7]. Likewise, case reports and prospective observations indicate that omalizumab therapy may restore tolerance to ASA and other COX‐1 inhibitors in a subset of patients [9]. These findings suggest that clinical reactivity in NECD is driven by variations in mast‐cell activation thresholds rather than fixed immunologic sensitization [3, 7]. Comparative results among different treatment strategies could help guide more informed therapeutic decisions in clinical practice.

This study aimed to evaluate the restoration of NSAID tolerance prospectively in adult patients with NECD and well‐controlled urticaria, to assess whether tolerance differs according to treatment status (omalizumab, regular antihistamines, or no treatment), and to determine the predictive value of the UCT and UAS7 for ASA provocation test outcomes.

## 2 Material and Methods

### 2.1 Study Design and Population

This single‐center prospective observational study was conducted at the Adult Immunology and Allergy Diseases Outpatient Clinic of Istanbul Faculty of Medicine. ASA and culprit NSAID provocation tests were performed prospectively as part of routine clinical evaluation, whereas skin prick test (SPT) and autologous serum skin test (ASST) results obtained before omalizumab initiation were retrieved retrospectively from medical records.

Adult patients (≥ 18 years) with CSU and a physician‐verified history compatible with NECD according to EAACI/ENDA criteria were eligible for inclusion [3]. Overall, 142 patients fulfilling these criteria were enrolled.

Patients were excluded for uncontrolled asthma, pregnancy or lactation, current angiotensin‐converting enzyme (ACE) inhibitor or β‐blocker use, unverified reaction history, incomplete clinical data, active infection, uncontrolled cardiovascular disease, anaphylaxis within the preceding 6 weeks, inability to suspend interacting medications, or any other contraindication to oral provocation.

Patients were assigned to three mutually exclusive groups at the index ASA provocation test: ongoing omalizumab for ≥ 3 months, regular antihistamines for ≥ 1 month without omalizumab, or no antihistamine use for ≥ 1 month and no previous omalizumab exposure. The overall timeline and group‐specific pathways are summarized in Supporting Information S1: Figure S1.

#### 2.1.1 Clinical and Laboratory Assessments

Demographic characteristics, comorbidities, personal and family history of allergic disease, and family history of CSU or NSAID hypersensitivity were recorded for all patients.

Disease‐related variables included age at first NSAID‐induced reaction, age at CSU onset, temporal relationship between CSU and NSAID reactions, disease durations, culprit drug class/specific agent, reaction phenotype. These prespecified variables were collected to characterize the NECD phenotype, describe baseline clinical heterogeneity, and examine their associations with ASA provocation outcomes as explanatory clinical variables rather than primary outcome measures.

Before ASA provocation, UCT [10], UAS7 [10], and the interval since the most recent CSU episode (time from the last self‐reported urticarial episode to the provocation test) were recorded. In the omalizumab‐treated group, age at treatment initiation and total treatment duration were also documented.

Laboratory tests at diagnosis included total IgE, peripheral blood eosinophil count, C‐reactive protein (CRP), erythrocyte sedimentation rate (ESR) and anti‐thyroid peroxidase antibody (anti‐TPO). ASST was performed by intradermal injection of 0.05 mL autologous serum into the volar forearm and read after 30 minutes; positivity was defined as a serum‐induced wheal ≥ 1.5 mm larger than the saline control [11].

In the omalizumab‐treated group, pretreatment SPT and ASST results were retrieved from records. For patients in the antihistamine‐treated and untreated groups, SPT and ASST were performed at study entry if no previous results were available in the medical records. SPT was read after 15 minutes and considered positive when the wheal was ≥ 3 mm larger than saline; atopy was defined as at least one positive aeroallergen SPT result [12].

#### 2.1.2 Group‐Specific Drug Provocation Strategy

Patients with documented NSAID hypersensitivity and well‐controlled CSU (UCT ≥ 12 and UAS7 ≤ 6) were eligible for provocation consistent with validated thresholds [10, 13].

Group‐specific procedures were as follows:

Omalizumab group: ASA provocation was performed without stopping clinically required omalizumab. The interval from the last injection to the provocation test was recorded, and culprit NSAID provocation was planned at 4 weeks if eligibility persisted.

Antihistamine group: ASA provocation was performed during regular antihistamine therapy. If negative, it was repeated 4 weeks after discontinuation. Positivity at either test defined ASA intolerance; two negative tests defined tolerance. Culprit NSAID provocation followed 4 weeks after the final ASA test.

Untreated group: ASA provocation was performed directly, followed 4 weeks later by culprit NSAID provocation regardless of the ASA result [14].

#### 2.1.3 Common Provocation Procedures

All oral drug provocation tests were performed in hospital at an EAACI Center of Excellence under institutional standard operating procedures (SOPs) covering eligibility, medication washout, dose escalation, objective stopping criteria, emergency treatment, and post‐challenge observation.

Except for prespecified on‐treatment testing, medications were withheld whenever clinically safe to reduce the risk of false‐negative results. In accordance with current drug provocation recommendations, H1 antihistamines, H2‐receptor antagonists, leukotriene receptor antagonists, and systemic corticosteroids were withheld for at least 7 days, 72 hours, 1 week, and 2 weeks, respectively [14, 15].

Provocation tests were single‐blind and placebo‐controlled: participants were blinded, whereas clinical assessors were not. Drugs and placebo were prepared in visually identical opaque capsules. Positivity required objective findings (≥ 3 new wheals, angioedema with pruritus, or systemic involvement); isolated subjective symptoms were not considered positive [3, 15].

ASA was administered every 30 minutes in doses of 50, 100, 150, 250, and 500 mg (cumulative 1050 mg). Testing was stopped when objective criteria were met, and the maximum dose was given only to patients without reactions at lower steps. Although 500 mg may be sufficient in many diagnostic settings, this pre‐existing institutional NECD protocol followed EAACI/Global Allergy and Asthma European Network (GA^2^LEN) guidance permitting cumulative doses of approximately 1000 mg in selected cutaneous NSAID hypersensitivity phenotypes [16]. The 1050‐mg cumulative dose reflected the predefined routine schedule and available tablet strengths and was not developed for the present study.

When clinically justified and accepted by the patient, culprit NSAID provocation was performed to assess drug‐specific tolerability for future individualized NSAID recommendations. Culprit drugs were selected from the reported index reaction and tested with stepwise protocols under the same safety conditions as the ASA challenge.

Reactions were managed according to the emergency protocol, including epinephrine, antihistamines, corticosteroids, oxygen, and intravenous fluids when indicated. Patients were observed for ≥ 6 h after the final dose, consistent with EAACI/GA^2^LEN recommendations and the typical timing of NECD reactions [16]; delayed reactions were monitored for up to 72 h by symptom diary and telephone follow‐up.

### 2.2 Outcome Measures

The primary outcome was patient‐level ASA provocation positivity by treatment group. Secondary outcomes were culprit NSAID provocation results, cumulative ASA dose at reaction, and clinical/laboratory predictors of ASA positivity.

### 2.3 Statistical Analysis

Analyses were performed using SPSS version 27 (IBM Corp., Armonk, NY, USA). Continuous variables are presented as median (interquartile range, IQR) or mean ± standard deviation, and categorical variables as *n* (%). Between‐group comparisons used *t*‐test or Mann–Whitney U, ANOVA or Kruskal–Wallis with post hoc tests, and Pearson chi‐squared, Fisher exact, or Fisher–Freeman–Halton tests as appropriate. ROC analysis assessed UCT, UAS7, and time since last CSU episode for predicting ASA positivity; optimal cut‐offs were identified using the Youden index. Area under the curve (AUC), 95% confidence interval (CI), sensitivity, specificity, positive predictive value (PPV), and negative predictive value (NPV) were reported. Because cut‐offs were derived from the present dataset and were not internally validated, they should be regarded as exploratory. Multivariable logistic regression identified independent predictors selected by clinical relevance and univariable associations, with results reported as odds ratios (ORs) and 95% CIs. Two‐sided *p* < 0.05 was considered statistically significant.

## 3 Results

### 3.1 Demographic and Clinical Characteristics of the Patients

A total of 142 patients with NECD were included in the study. The median age was 40 years (IQR: 31.5–52), and 79.6% were female. The median duration of CSU was 4 years (IQR, 2–8). NSAID reactions most frequently occurred after CSU onset (52.1%), and the most common clinical presentation was combined urticaria and angioedema (64.1%). The demographic and clinical characteristics of the study population are summarized in Table 1.

**TABLE 1:** Demographic and clinical characteristics of the patients (*n* = 142).

Characteristic	Value
Age, years, median (IQR)	40 (31.5–52)
Female sex, *n* (%)	113 (79.6)
Comorbidity, *n* (%)	94 (66.2)
Personal history of allergic disease, *n* (%)	58 (40.8)
Drug allergy other than NSAID, *n* (%)	27 (19.0)
Family history of allergic disease, *n* (%)	62 (43.7)
Family history of NSAID hypersensitivity, *n* (%)	8 (5.6)
Family history of CSU, *n* (%)	23 (16.2)
Age at first NSAID reaction, years, median (IQR)	35 (26–45)
Age at CSU diagnosis, years, median (IQR)	33.5 (25–44)
Duration of CSU, years, median (IQR)	4 (2–8)
Timing of NSAID reaction relative to CSU onset, *n* (%)	
Before CSU onset	20 (14.1)
After CSU onset	74 (52.1)
Simultaneous with CSU onset	48 (33.8)
Index NSAID reaction, *n* (%)	
Urticaria and angioedema	91 (64.1)
Urticaria alone	39 (27.5)
Angioedema alone	12 (8.5)
Atopy (SPT positive), *n* (%)	26 (18.3)
ASST positivity, *n* (%)	26 (18.3)

*Note:* Data are shown as median (IQR) or *n* (%), unless otherwise indicated. Percentages use the full cohort (*n* = 142) as the denominator.

Abbreviations: ASST, autologous serum skin test; CSU, chronic spontaneous urticaria; IQR, interquartile range; NSAID, nonsteroidal anti‐inflammatory drug; SPT, skin prick test.

Aryl‐propionic acids were the predominant culprits (74.6%) followed by diclofenac (17.6%), acetylsalicylic acid (16.9%), and paracetamol (12.6%), while pyrazolone derivatives such as metamizole were less frequent but clinically relevant (Table 2).

**TABLE 2:** Prevalence of NSAID hypersensitivity reactions by chemical category.

	Patients, *n* (%)
Salicylic acid derivatives	24 (16.9)
Acetylsalicylic acid	24 (16.9)
Indole and indene acetic acids	2 (1.4)
Indomethacin	1 (0.7)
Etodolac	2 (1.4)
Hetero‐aryl acetic acids	25 (17.6)
Diclofenac	25 (17.6)
Aryl‐propionic acids	106 (74.6)
Flurbiprofen	35(24.6)
Ibuprofen	39(27.5)
Ketoprofen	45(31.7)
Naproxen	17(12.0)
Pyrazolone derivatives	9(6.3)
Metamizole	9 (6.3)
Propyphenazone	1(0.7)
Para‐aminophenol derivates	18 (12.6)
Paracetamol	18 (12.6)

*Note:* Patients could report more than one culprit NSAID; therefore percentages may exceed 100%. Chemical‐class totals indicate unique patients with at least one culprit in that class and may be lower than the sum of individual agents, because repeated agents within a class were counted once.

### 3.2 Comparison of Clinical Features of the Patients Across Different Treatment Modalities

Selected clinical characteristics and ASA provocation outcomes according to treatment group are summarized in Table 3. Overall, ASA provocation was positive in 29 of 142 patients (20.4%). ASA provocation positivity was significantly higher in the antihistamine‐treated group (38.9%) than in the omalizumab‐treated (11.5%; *p* = 0.001) and untreated groups (3.7%; *p* < 0.001), whereas no significant difference was observed between the omalizumab‐treated and untreated groups (*p* = 0.43). In the antihistamine‐treated group, 16 patients were positive during the initial on‐treatment challenge, and 5 additional patients became positive after antihistamine withdrawal. Compared with the antihistamine‐treated group, the omalizumab‐treated group had a longer duration of CSU and a longer interval since the last CSU episode, lower daily antihistamine use, fewer NSAID‐induced reactions, higher UCT scores, and lower UAS7 scores (all *p* ≤ 0.017). The antihistamine‐treated group also had a shorter interval since the last CSU episode, lower UCT scores, and higher UAS7 scores than the untreated group (all *p* < 0.001). Variables without significant differences among treatment groups are presented in Supporting Information S3: Table S1.

**TABLE 3:** Clinical characteristics and ASA provocation outcomes according to treatment group.

	(1) Omalizumab‐treated (*n* = 61)	(2) Antihistamine‐treated (*n* = 54)	(3) Untreated (*n* = 27)	*p* (1–2)	*p* (2–3)
ASA provocation test positivity, *n* (%)	7 (11.5)	21 (38.9)	1 (3.7)	*p* = 0.001	*p* < 0.001
Duration of CSU, years, median (IQR)	6 (4–10)	3 (2–6)	2 (2–4)	*p* < 0.001	NS
Interval since the last CSU episode, months, median (IQR)	7 (3–12)	1 (1–2)	12 (7–24)	*p* < 0.001	*p* < 0.001
Daily number of antihistamine tablets, median (IQR)	1 (1–2)	2 (1–2)	—	*p* = 0.017	—
Number of NSAID‐induced reactions, median (IQR)	1 (1–2)	2 (1–2)	2 (1–2)	*p* = 0.002	NS
UCT before ASA provocation, median (IQR)	16 (16–16)	14 (13–15)	16 (16–16)	*p* < 0.001	*p* < 0.001
UAS7 before ASA provocation, median (IQR)	0 (0–3)	3 (1–4)	0 (0–2)	*p* < 0.001	*p* < 0.001

*Note:* Data are shown as median (IQR) or *n* (%). Groups are defined by treatment status at the index ASA provocation test. In the antihistamine‐treated group, ASA positivity was defined by either the on‐treatment or post‐withdrawal challenge. Only significant or outcome‐relevant variables are shown; other baseline variables are in Supporting Information S3: Table S1.

Abbreviations: —, not applicable; ASA, acetylsalicylic acid; CSU, chronic spontaneous urticaria; IQR, interquartile range; NS, not significant; UAS7, Urticaria Activity Score over 7 days; UCT, Urticaria Control Test.

### 3.3 Baseline Clinical and Laboratory Characteristics According to ASA Provocation Results in the Overall Cohort and Across Treatment Groups

Clinical and laboratory characteristics according to ASA provocation outcome are presented in Table 4. In the overall cohort, ASA‐positive patients had a shorter CSU duration and a shorter interval since the last CSU episode, lower UCT scores and CRP levels, and higher UAS7 scores than ASA‐negative patients (all *p* ≤ 0.014) (Figure 1). A family history of NSAID hypersensitivity, atopy, and ASST positivity were also more frequent among ASA‐positive patients (*p* = 0.009, *p* = 0.047, and *p* = 0.002, respectively).

**TABLE 4:** Characteristics according to ASA provocation outcome, overall and by treatment group.

	ASA provocation test results in all patients			ASA provocation outcomes in the omalizumab group			ASA provocation outcomes in the antihistamine‐treated group		
	ASA provocation test positive (*n* = 29)	ASA provocation test negative (*n* = 113)	*p*	ASA provocation test positive (*n* = 7)	ASA provocation test negative (*n* = 54)	*p*	ASA provocation test positive (*n* = 21)	ASA provocation test negative (*n* = 33)	*p*
Family history of NSAID hypersensitivity, *n* (%)	5 (17.2)	3 (2.7)	*p* = 0.009	1(14.3)	3(5.6)	NS	4 (19.0)	0 (0.0)	*p* = 0.019
Duration of CSU, years, median (IQR)	3 (1–6)	5 (3–8)	*p* = 0.014	—	—	—	—	—	—
Interval since the last CSU episode, months, median (IQR)	1 (1–1)	6 (2–12)	*p* < 0.001	1 (1–2)	8 (5–12)	*p* = 0.003	1 (1–1)	1 (1–3)	NS
UCT before ASA provocation, median (IQR)	14 (14–16)	16 (16–16)	*p* < 0.001	13 (12–14)	16 (16–16)	*p* = 0.001	14 (14–15)	16 (14–16)	*p* = 0.004
UAS7 before ASA provocation, median (IQR)	4 (3–5)	0 (0–2)	*p* < 0.001	4 (4–5)	0 (0–2)	*p* = 0.001	4 (3–5)	2 (0–3)	*p* = 0.001
Atopy (SPT positive), *n* (%)	9 (31.0)	17 (15.0)	*p* = 0.047	1 (14.3)	11 (20.4)	NS	8 (38.1)	4 (12.1)	*p* = 0.042
ASST positivity, *n* (%)	11 (37.9)	15 (13.3)	*p* = 0.002	3(42.9)	5(9.3)	*p* = 0.042	8 (38.1)	6 (18.2)	NS
CRP, mg/L, median (IQR)	2 (1–5)	5 (2–7)	*p* = 0.004	6 (4–13)	5.5 (3–7)	NS	2 (0–4)	5 (3–6)	*p* = 0.003
Cumulative ASA dose at positivity, mg, median (IQR)	1050 (550–1050)	—	—	1050 (550–1050)	—	—	1050 (550–1050)	—	—
Time to reaction during ASA provocation, min, median (IQR)	120 (50–360)	—	—	240 (80–360)	—	—	110 (48–302)	—	—

*Note:* Only variables significant in at least one comparison or directly relevant to the outcome are shown. Non‐associated variables are listed in Supporting Information S4: Table S2, and omalizumab‐specific variables in Supporting Information S5: Table S3. The untreated group is not shown separately because only one patient was ASA‐positive.

Abbreviations: —, not applicable; ASA, acetylsalicylic acid; ASST, autologous serum skin test; CRP, C‐reactive protein; CSU, chronic spontaneous urticaria; IQR, interquartile range; NS, not significant; NSAID, nonsteroidal anti‐inflammatory drug; SPT, skin prick test; UAS7, Urticaria Activity Score over 7 days; UCT, Urticaria Control Test.

**FIGURE 1:**
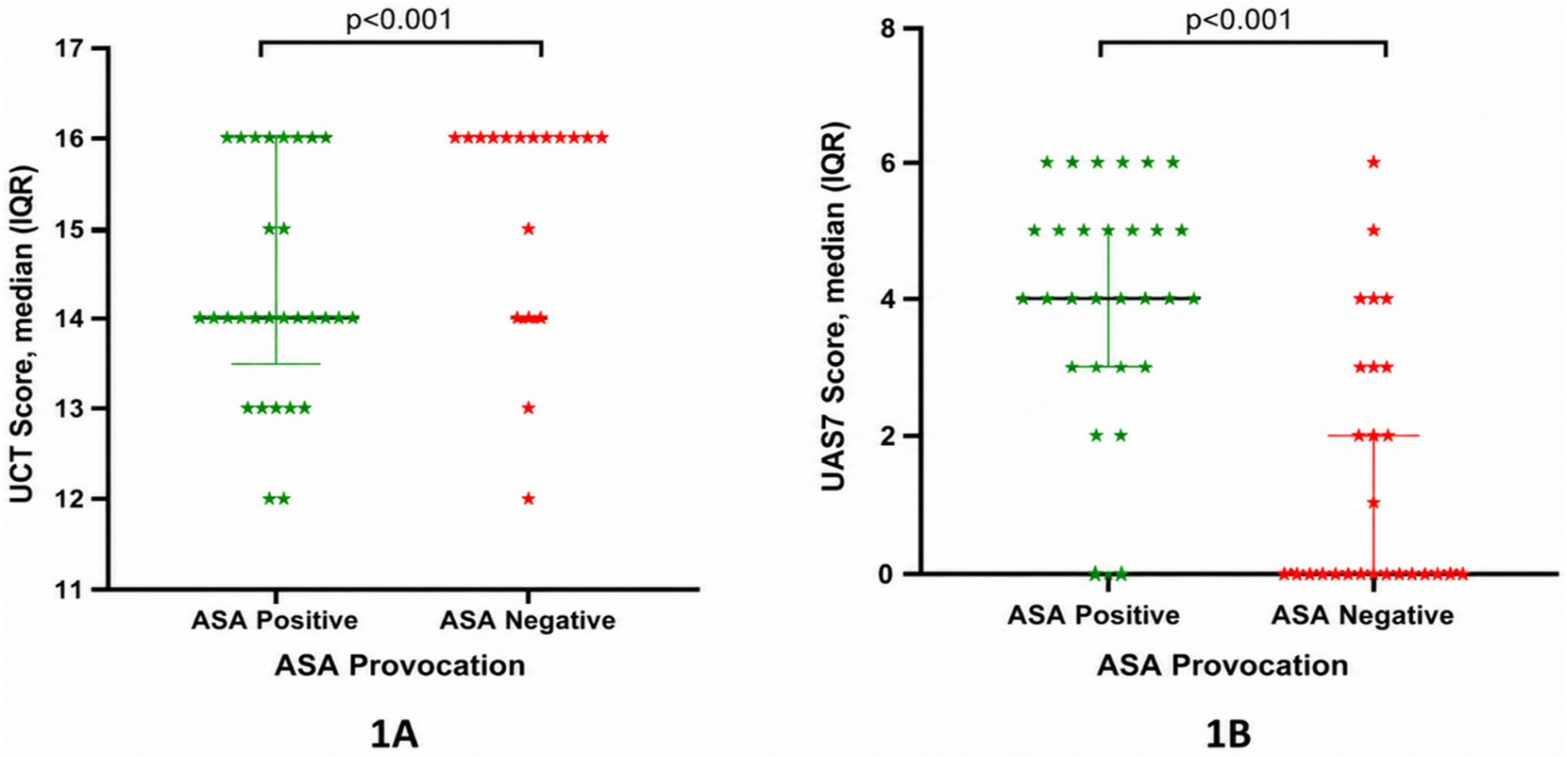
UCT (A) and UAS7 (B) scores by ASA provocation outcome. ASA, Acetylsalicylic acid; UAS7, Urticaria Activity Score over 7 days; UCT, Urticaria Control Test.

Within the omalizumab‐treated group, ASA‐positive patients had a shorter interval since the last CSU episode together with lower UCT scores and higher UAS7 scores (all *p* ≤ 0.003), and ASST positivity was more frequent (*p* = 0.042). Within the antihistamine‐treated group, ASA‐positive patients more frequently had a family history of NSAID hypersensitivity and atopy and had lower UCT scores and CRP levels and higher UAS7 scores (all *p* ≤ 0.042). No separate subgroup analysis was performed for the untreated group because only one patient was ASA‐positive.

Variables not associated with ASA provocation outcome are presented in Supporting Information S4: Table S2. Among omalizumab‐treated patients, concomitant antihistamine use was more frequent in the ASA‐positive subgroup than in the ASA‐negative subgroup (85.7% vs. 16.7%; *p* = 0.001) The remaining omalizumab‐related variables did not differ according to ASA provocation outcome (Supporting Information S5: Table S3).

### 3.4 UCT and UAS7 Scores by ASA Provocation Status

The median (IQR) UCT scores differed between patients with positive and negative ASA provocation tests, with a median UCT score of 14(14–16) in ASA provocation positive patients compared with 16(16–16) in provocation negative patients (Figure 1A). Similarly, the median (IQR) weekly UAS7 values varied according to ASA provocation status, with a median UAS7 score of 4(3–5) in ASA provocation positive patients and 0(0–2) in provocation negative patients (Figure 1B).

### 3.5 ROC Analysis of UCT and UAS7 in Predicting ASA Provocation Outcomes

The ROC analysis demonstrated that UCT had good discriminatory ability for predicting ASA provocation positivity, with an optimal cut‐off value of UCT ≤ 15, yielding a sensitivity of 72.4%, specificity of 83.2%, and an AUC of 0.792. UAS7 showed excellent discriminatory performance, with an optimal cut‐off value of UAS7 ≥ 4, providing a sensitivity of 72.4%, specificity of 86.7%, and an AUC of 0.857. The corresponding negative predictive values were 92.2% and 92.5%, respectively. Overall, both UCT ≤ 15 and UAS7 ≥ 4 were identified as strong predictors of ASA provocation positivity (Figure 2A,B).

**FIGURE 2:**
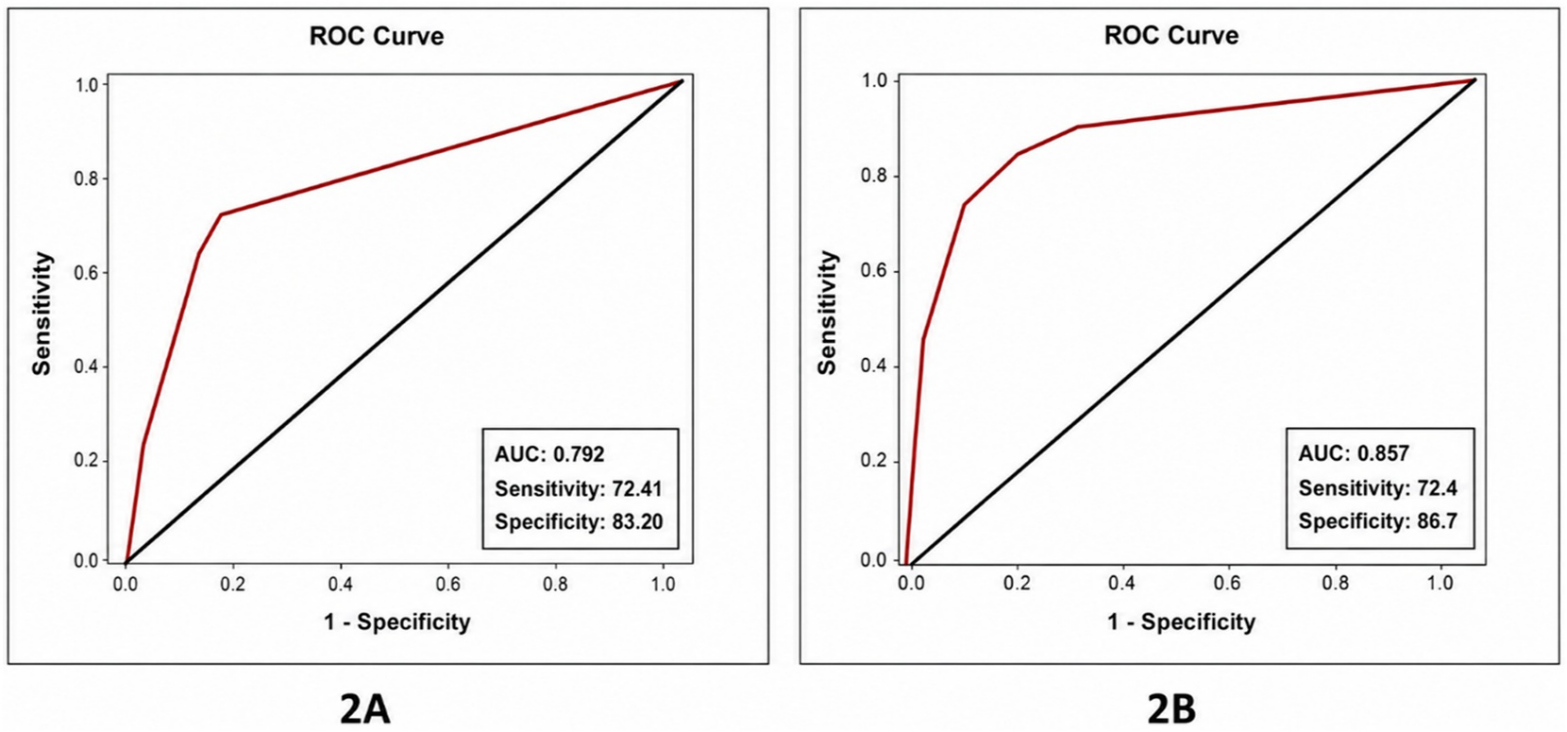
ROC curves for UCT (A) and UAS7 (B) in predicting ASA provocation positivity. ASA, Acetylsalicylic acid; ROC, Receiver operating characteristic; UAS7, Urticaria Activity Score over 7 days; UCT, Urticaria Control Test.

### 3.6 Multivariable Analysis

Logistic regression included family history of NSAID hypersensitivity, duration of CSU, interval since the last CSU episode, UCT, UAS7, atopy, ASST positivity, and CRP. The final model (Nagelkerke *R* ^2^ = 52.8%) retained two independent predictors: a longer interval since the last CSU episode was associated with lower odds of positivity (OR 0.728; 95% CI 0.556–0.952; *p* = 0.020), whereas a higher UAS7 was associated with higher odds (OR 1.708; 95% CI 1.268–2.300; *p* < 0.001) (Table 5).

**TABLE 5:** Logistic regression analysis of risk factors affecting ASA provocation positivity.

	*p*	OR	95% CI	
			Lower	Upper
Interval since the last CSU episode (months)	0.020	0.728	0.556	0.952
UAS7 before ASA provocation	< 0.001	1.708	1.268	2.300
CRP (mg/L)	0.062	0.851	0.719	1.008

*Note:* Nagelkerke *R* ^2^ = 0.528.

Abbreviations: ASA, Acetylsalicylic acid; CI, Confidence interval; CRP, C‐reactive protein; CSU, Chronic spontaneous urticaria; UAS7, Urticaria Activity Score over 7 days.

### 3.7 Predictive Value of Time Since the Last CSU Episode for ASA Provocation Test Positivity

The predictive value of the time elapsed since the most recent CSU episode for ASA provocation test positivity was evaluated using ROC curve analysis. The analysis identified a cut‐off value of ≤ 2 months as the optimal threshold for discriminating between ASA provocation positive and negative patients. At this cut off, the sensitivity was 96.55%, specificity 69.91%, positive predictive value 45.20%, and negative predictive value 98.70%. The area under the ROC curve (AUC) was 0.833 (95% confidence interval [CI]: 0.762–0.890), indicating good discriminatory performance. A statistically significant association was observed between a time interval of ≤ 2 months since the last CSU episode and ASA provocation positivity (*p* = 0.001). Patients who had experienced a CSU episode within the preceding 2 months had a markedly higher likelihood of a positive ASA provocation test, with an odds ratio (OR) of 65.06 (95% CI: 8.50–497.73), although the wide confidence interval reflects the limited number of positive challenges (Figure 3).

**FIGURE 3:**
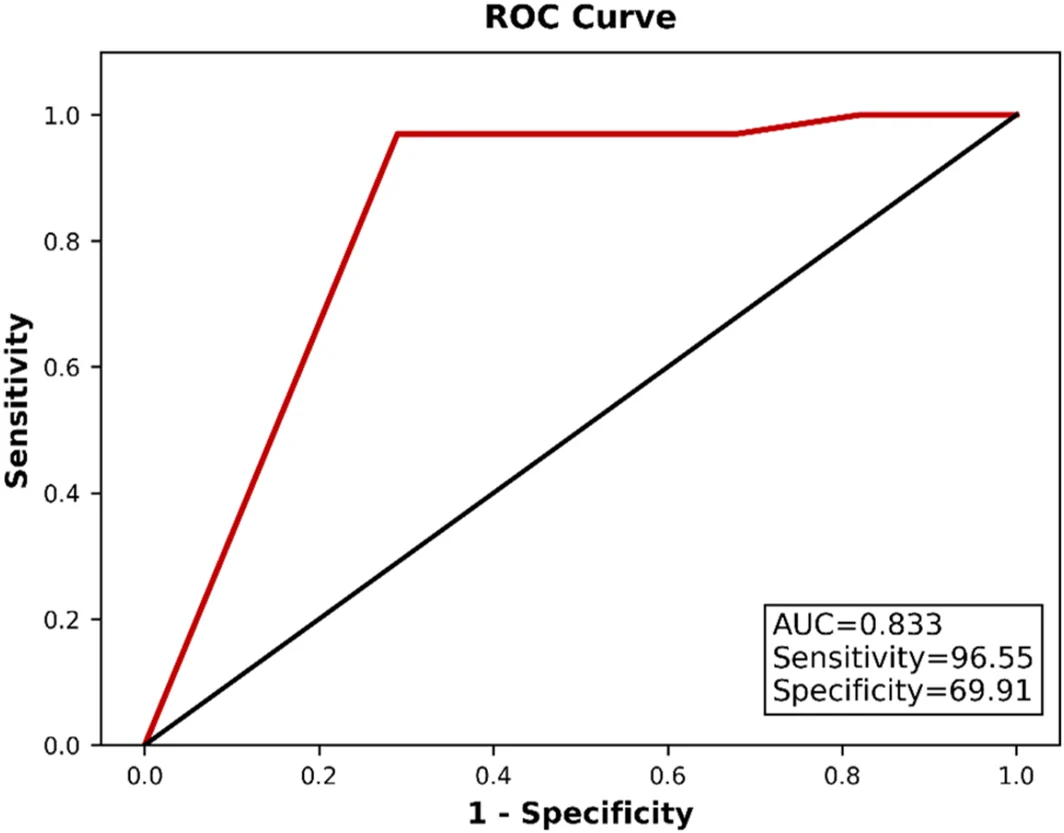
ROC curve for time since the most recent CSU episode in predicting ASA provocation positivity. ASA, acetylsalicylic acid; CSU, chronic spontaneous urticaria; ROC, receiver operating characteristic.

### 3.8 ASA and Drug Provocation Test Results

Patient flow and provocation outcomes by treatment group are summarized in Figure 4. Culprit drug provocation testing was completed in 130 of 142 patients; 12 patients refused the test. Most challenges were negative (97.7%), and all positive culprit‐drug challenges occurred in ASA‐positive patients.

**FIGURE 4:**
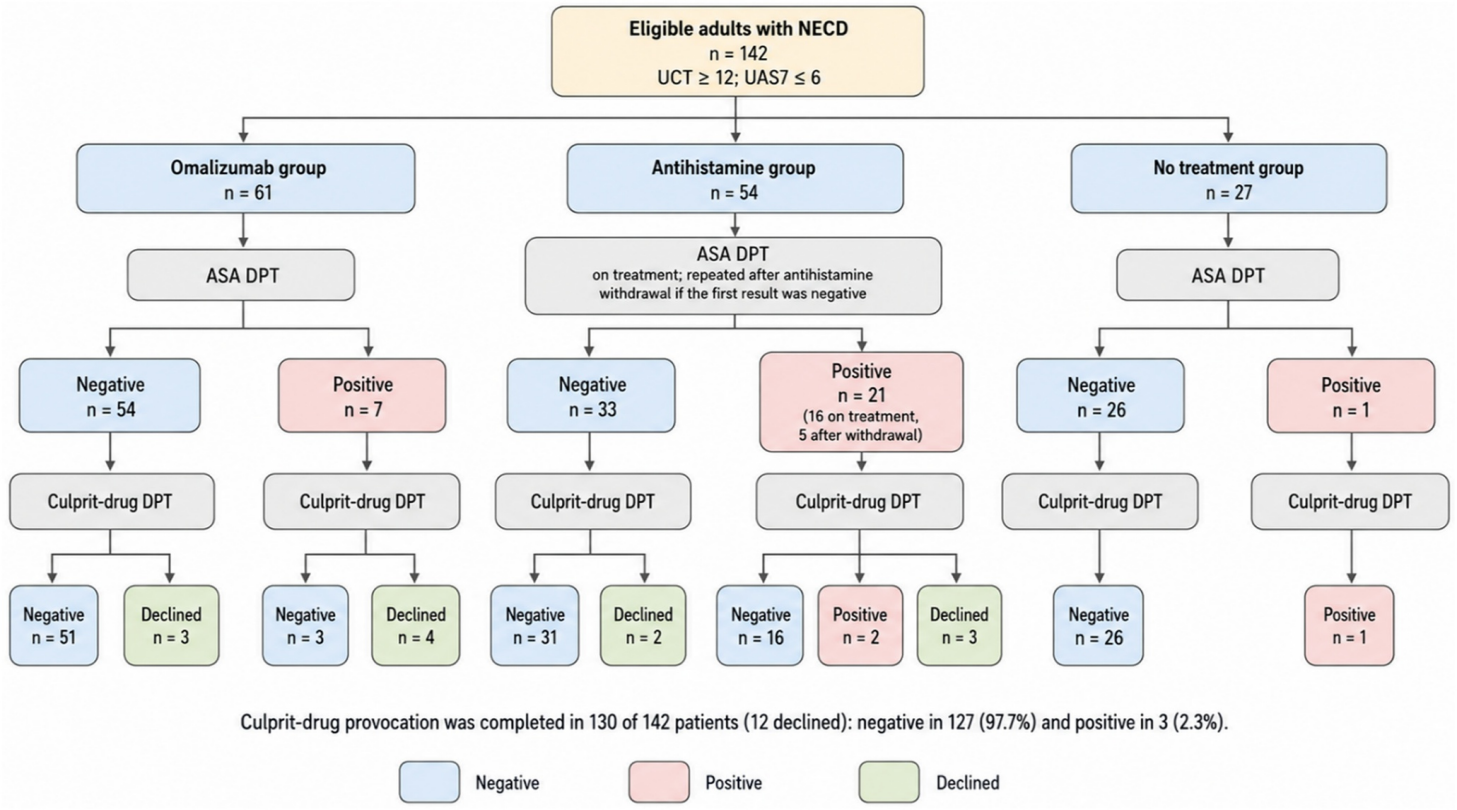
Study flow and ASA/culprit‐drug provocation outcomes by treatment group.

Eligible patients were classified according to treatment status and underwent protocol‐defined ASA provocation testing. In the antihistamine‐treated group, ASA positivity was determined by either the initial challenge performed during antihistamine treatment or the repeat challenge after antihistamine withdrawal. Patients subsequently underwent culprit‐drug provocation according to the predefined protocol or declined testing.

## 4 Discussion

This study clarified the dynamics of NSAID tolerance in NECD by evaluating a large cohort of patients with well controlled CSU using ASA provocation tests and validated disease activity instruments, including UCT and UAS7. The comparison of multiple treatment groups including patients receiving omalizumab, antihistamines, or no treatment further enabled a comprehensive evaluation of treatment specific effects on NSAID tolerance. Most importantly, our study demonstrated that NSAID tolerance in NECD can be regained not only during remission but also when CSU is adequately controlled, even without complete suppression of disease activity. This finding challenges the traditional approach advocating permanent or indefinite avoidance of NSAIDs in NECD and highlights the potential for adopting more flexible, control based personalized strategies in clinical practice, thereby offering valuable insight to existing literature.

CSU remission is typically defined as a period of at least 6 months without medication and free of symptoms such as urticaria, angioedema, and pruritus [6]. In the prospective cohort study by Sánchez et al., remission was evaluated according to these strict criteria, and remission, recurrence, and persistent disease trajectories were comprehensively characterized over a 5 year follow up period [17]. Strict remission criteria require prolonged drug free periods, which may not always be feasible for NECD patients and may lead to unnecessary long‐term avoidance of NSAIDs particularly in individuals for whom NSAID therapy may be clinically indicated, such as those with coronary artery disease or rheumatologic conditions. Furthermore, although definitions of remission have been proposed for CSU, their direct application to NECD may not be appropriate, as NSAID intolerance represents a key and defining component of this condition. For these reasons, our study did not adhere rigidly to a remission‐based definition; instead, NSAID tolerance was evaluated according to a state of well control, defined by UCT ≥ 12 and UAS7 ≤ 6. This approach demonstrated quantitatively that NSAID tolerance may be regained even in active, yet well controlled, CSU without the need to wait for full remission. Although these findings do not imply that NSAIDs can be safely reintroduced in all patients, they provide a rationale for a more flexible, clinically contextualized decision‐making framework. In this way, the model we propose may help reduce unnecessary avoidance of NSAIDs while still prioritizing patient safety and individual therapeutic needs.

In our study, the lower UCT scores and higher UAS7 values observed in the antihistamine group indicate insufficient control of CSU in these patients. In contrast, the higher UCT and lower UAS7 scores in the omalizumab and untreated group suggest more stable and better controlled disease. These differences in disease control are consistent with the distribution of ASA provocation outcomes: positivity was highest in the antihistamine group (38.9%) lower in the omalizumab group (11.5%), and almost negligible in the untreated group (3.7%). This gradient followed disease stability rather than treatment itself: many untreated patients had been symptom‐free long enough to meet formal remission criteria, whereas the omalizumab group had controlled but ongoing disease yet still showed high tolerance. This is the central finding of our study. Our findings align with the data reported by Sánchez et al., demonstrating that, once adequate clinical control is achieved under antihistamine therapy, NSAID tolerance can be restored in approximately 75% of patients who previously reacted to these agents [7]. Collectively, these results quantitatively support the concept that NSAID reactions in NECD represent a dynamic process largely driven by the underlying level of CSU activity.

The efficacy and safety of omalizumab in antihistamine refractory CSU have been well established in randomized controlled trials [18]. In the context of NSAID hypersensitivity, previous case reports and small patient series have shown that ASA tolerance may be restored during omalizumab therapy [9], and studies conducted in NSAID‐exacerbated respiratory disease (NERD) populations similarly demonstrate that omalizumab can reestablish ASA tolerance in a substantial proportion of patients [19]. Furthermore, prospective evidence indicating that NSAID‐induced cutaneous reactions improve in parallel with CSU remission supports the notion that NSAID hypersensitivity is closely linked to underlying disease activity [17]. Our study is the first to prospectively evaluate these observations in the largest NECD cohort treated with omalizumab to date, demonstrating that 88.5% of the patients exhibited negative ASA provocation results, indicative of high NSAID tolerance under therapy. The fact that positive provocation outcomes were confined to a small subgroup characterized by persistent CSU activity further suggests that hypersensitivity is driven by inadequate disease control rather than by the presence or absence of omalizumab itself. Collectively, these findings provide the first quantitative, large‐scale confirmation that achieving sufficient disease control with omalizumab can substantially enhance NSAID tolerance in NECD.

In our study, UCT and UAS7 not only differentiated disease control levels across treatment groups but also demonstrated high accuracy in predicting ASA provocation test results. The ROC analysis showed that the thresholds of UCT ≤ 15 and UAS7 ≥ 4 yielded strong sensitivity, specificity, and particularly high negative predictive values, indicating their clinical utility in ruling out ASA positivity. Although the association between increased CSU activity and NSAID reactions in the NECD phenotype is well recognized, this relationship has not previously been defined using quantitative thresholds [20]. Our findings provide the first evidence that, without requiring remission, the thresholds of UCT ≤ 15 and UAS7 ≥ 4 can reliably predict ASA provocation outcomes during active yet well controlled CSU, representing a novel and meaningful contribution to the literature. However, when multiple clinical variables were evaluated simultaneously, only UAS7 and the time elapsed since the last CSU episode remained independently associated with ASA provocation positivity, whereas UCT did not retain independent statistical significance. This discrepancy may be attributable to the different constructs captured by these instruments: while UCT reflects overall disease control over the preceding 4 weeks and incorporates patient perception, UAS7 provides a more granular and quantitative assessment of urticaria activity, potentially capturing residual or subclinical disease activity that may be relevant to NSAID hypersensitivity.

The EAACI/GA^2^LEN guidelines emphasize that NSAID hypersensitivity reactions are more likely to occur during periods of active disease and may become less pronounced once adequate disease control or remission is achieved; however, they do not define a specific temporal threshold [5, 15]. Some studies suggest that NSAID tolerance may be associated with clinical control; however, they do not define a standardized symptom free interval threshold [7, 21]. In our study, ASA provocation outcomes were more closely associated with recent disease activity than with overall disease duration. Accordingly, the antihistamine group characterized by the shortest interval since the most recent CSU episode exhibited the highest rate of ASA positivity, whereas longer symptom free intervals, observed in the omalizumab group and even more prominently in the untreated group, were associated with lower provocation positivity. Importantly, ROC curve analysis identified a time interval of ≤ 2 months since the last CSU episode as a clinically meaningful cut off, demonstrating strong discriminatory performance and a very high negative predictive value. The markedly increased likelihood of ASA positivity among patients with recent disease activity further supports the concept that temporal disease stability at the time of assessment, independent of disease chronicity, represents a key determinant of ASA reactivity. Our study not only reinforces existing guideline recommendations but also provides novel quantitative evidence; however, this proposed time based threshold should be validated in independent cohorts before broader implementation in clinical practice.

The association between ASA provocation positivity and a positive family history of NSAID hypersensitivity observed in our study is consistent with previous reports suggesting a genetic predisposition to NSAID hypersensitivity [22]. Atopy was likewise more frequent among ASA‐positive patients, both in the overall cohort and within the antihistamine‐treated group, although its clinical relevance remains uncertain. In addition, prior studies have shown that, particularly in autoimmune endotypes of CSU, ASST positivity and elevated CRP levels are associated with greater disease severity [11, 23]. ASST positivity was more frequent among provocation‐positive patients in our cohort, consistent with these reports. In contrast, and unexpectedly, CRP levels were lower in provocation‐positive patients; this may reflect the narrow range of systemic inflammatory activity in a uniformly well‐controlled population. However, these variables did not retain statistical significance in the multivariable regression model, suggesting that these associations may be weak or indirect and largely mediated by other factors, such as overall disease activity and temporal characteristics.

The distribution of reported culprit NSAIDs in our cohort, predominantly aryl‐propionic acid derivatives, is consistent with previous literature, whereas the frequency of pyrazolone derivatives such as metamizole may partly reflect regional prescribing patterns [3]. Culprit NSAID provocation was performed to assess drug‐specific clinical tolerability and guide individualized future NSAID recommendations. Although tolerance of the culprit NSAID in ASA‐positive patients may appear unexpected, this finding is consistent with the dynamic nature of NECD. In the antihistamine‐treated group, 16 of 21 ASA‐positive patients (76.2%) tolerated the culprit NSAID, closely paralleling the findings of Sánchez et al., who reported tolerance in 72 of 96 patients (75%) when CSU was clinically controlled with antihistamines [7]. These findings suggest that ASA‐positive/culprit‐negative results reflect drug‐specific clinical tolerance under controlled conditions rather than complete resolution of COX‐1 cross‐reactivity, probably owing to an increased reaction threshold associated with improved CSU control. Conversely, positive culprit NSAID reactions occurred only in ASA‐positive patients, supporting the role of residual disease activity in persistent NSAID hypersensitivity. Therefore, culprit NSAID provocation may provide useful, individualized information, although tolerance to the tested drug should not be generalized to unrestricted use of all strong COX‐1 inhibitors.

The study has several limitations. Its single‐center design and relatively homogeneous population may limit generalizability. Medication status differed across groups: initially negative patients in the antihistamine group were retested off therapy, whereas omalizumab and concomitant antihistamines were continued, potentially suppressing cutaneous reactions; between‐group positivity rates may therefore reflect both treatment effects and medication status. Because inclusion required well‐controlled disease, UCT and UAS7 varied within narrow ranges, and the cut‐offs were derived from the present dataset without internal validation; they should not be extrapolated to poorly controlled CSU. The limited number of positive challenges also constrained the multivariable analysis, and the absence of longitudinal follow‐up precluded assessment of long‐term tolerance. Nevertheless, the large sample size, prospective placebo‐controlled design, and comparison across treatment strategies strengthen the study.

## 5 Conclusion

In this prospective study, we showed that a substantial proportion of patients with NECD and well‐controlled CSU can regain NSAID tolerance on ASA provocation testing, even in the absence of complete remission. Our findings indicate that the primary determinant of NSAID reactivity is the level of disease activity at the time of testing. In ROC analyses, the thresholds of UCT ≤ 15 and UAS7 ≥ 4, as well as an interval of ≤ 2 months since the last CSU episode, emerged as clinically applicable cut‐offs for discriminating ASA provocation positivity. In multivariable analysis, UAS7 and the time elapsed since the last CSU episode remained independently associated with ASA positivity. These results suggest that, with appropriate patient selection and under controlled conditions, provocation testing may facilitate the safe reintroduction of NSAIDs while reducing unnecessary NSAID avoidance in NECD. The proposed cut‐offs should be validated in independent, multicenter cohorts, and the long‐term persistence of regained tolerance warrants further investigation.

## Author Contributions

**Merve Hormet Igde:** writing – original draft, conceptualization, methodology, data curation, formal analysis, validation, supervision, investigation, writing – review and editing. **Pelin Korkmaz:** conceptualization, data curation, investigation, methodology, validation. **Semra Demir:** writing – review and editing, supervision, validation, investigation, conceptualization. **Osman Ozan Yegit:** conceptualization, investigation, writing – review and editing, supervision. **Derya Ünal:** conceptualization, investigation, writing – review and editing, supervision. **Merve Dilşad Atasever:** data curation, formal analysis. **Ayşe Feyza Aslan:** data curation, formal analysis, investigation. **Leyla Bölek:** investigation, formal analysis, data curation. **Selin Ece Güzel:** investigation, data curation, methodology. **Nevzat Kahveci:** data curation, investigation. **Mehmet Emin Sezgin:** investigation, data curation. **Şule Çelik Kamacı:** data curation. **Esra Kaya:** data curation. **Bircan Erden:** data curation. **Asli Gelincik:** writing – review and editing, conceptualization, data curation, investigation, methodology, validation, visualization, formal analysis, supervision,

## Funding

The authors have nothing to report.

## Ethics Statement

Written informed consent was obtained from all patients. The study was approved by the Istanbul Faculty of Medicine Clinical Research Ethics Committee (approval number: 2024/2432; approval date: 21 February 2025; meeting no. 04; official document no. E‐29624016‐050.99‐3220088) and was conducted in accordance with the Declaration of Helsinki.

## Conflicts of Interest

The authors declare no conflicts of interest.

## Supporting information


Supporting Information S1

**Figure S1:** Study Timeline and Data Collection Framework.

**Table S1:** Baseline variables that did not differ between treatment groups.

**Table S2:** Variables not associated with ASA provocation outcome.

**Table S3:** Omalizumab treatment variables according to ASA provocation outcome (omalizumab‐treated group, *n* = 61).

## Data Availability

The data that support the findings of this study are available from the corresponding author upon reasonable request.
