# Supplementary material for: NSAID Tolerance Can Be Restored in NECD Even in the Absence of Complete Remission of Urticaria
Source: Clin Transl Allergy. 2026 Oct 21;16(10):e70216. doi: 10.1002/clt2.70216 (PMC13687787; doi:10.1002/clt2.70216)
Supplement: Supplementary file 1 [file CLT2-16-e70216-s001.docx]

**Supplementary Table S1. Baseline variables that did not differ between treatment groups.**

|  | **(1) Omalizumab-treated (n = 61)** | **(2) Antihistamine-treated (n = 54)** | **(3) Untreated (n = 27)** | **p (1–2)** | **p (2–3)** |
| --- | --- | --- | --- | --- | --- |
| Age, years, mean ± SD | 42.38 ± 14.09 | 41.61 ± 14.05 | 42.78 ± 14.82 | NS | NS |
| Female sex, n (%) | 52 (85.2) | 42 (77.8) | 19 (70.4) | NS | NS |
| Current smokers, n (%) | 16 (26.2) | 13 (24.1) | 4 (14.8) | NS | NS |
| Comorbidity, n (%) | 45 (73.8) | 35 (64.8) | 14 (51.9) | NS | NS |
| Family history of allergic disease, n (%) | 26 (42.6) | 23 (42.6) | 13 (48.1) | NS | NS |
| Family history of NSAID hypersensitivity, n (%) | 4 (6.6) | 4 (7.4) | 0 (0.0) | NS | NS |
| Family history of CSU, n (%) | 8 (13.1) | 10 (18.5) | 5 (18.5) | NS | NS |
| Age at first NSAID reaction, years, mean ± SD | 35.30 ± 14.67 | 36.91 ± 13.96 | 37.70 ± 14.90 | NS | NS |
| Age at CSU diagnosis, years, mean ± SD | 33.67 ± 13.71 | 36.15 ± 14.01 | 37.44 ± 14.60 | NS | NS |
| Interval between CSU onset and first NSAID reaction, months, median (IQR) | 24 (12–60) | 24 (12–48) | 12 (12–48) | NS | NS |
| Atopy (SPT positive), n (%) | 12 (19.7) | 12 (22.2) | 2 (7.4) | NS | NS |
| ASST positivity, n (%) | 8 (13.1) | 14 (25.9) | 4 (14.8) | NS | NS |

*Data are shown as mean ± SD, median (IQR), or n (%), as appropriate. Groups are defined in Table 3. ASST, autologous serum skin test; CSU, chronic spontaneous urticaria; IQR, interquartile range; NS, not significant; NSAID, nonsteroidal anti-inflammatory drug; SD, standard deviation; SPT, skin prick test.*
