# Supplementary material for: NSAID Tolerance Can Be Restored in NECD Even in the Absence of Complete Remission of Urticaria
Source: Clin Transl Allergy. 2026 Oct 21;16(10):e70216. doi: 10.1002/clt2.70216 (PMC13687787; doi:10.1002/clt2.70216)
Supplement: Supplementary file 2 [file CLT2-16-e70216-s002.docx]

**Supplementary Table S2. Variables not associated with ASA provocation outcome.**

|  | **ASA Provocation Test Results in All Patients** | | | **ASA Provocation Outcomes in the Omalizumab Group** | | | **ASA Provocation Outcomes in the Antihistamine-Treated Group** | | |
| --- | --- | --- | --- | --- | --- | --- | --- | --- | --- |
|  | **ASA provocation test positive (n = 29)** | **ASA provocation test negative (n = 113)** | **p** | **ASA provocation test positive (n = 7)** | **ASA provocation test negative (n = 54)** | **p** | **ASA provocation test positive (n = 21)** | **ASA provocation test negative (n = 33)** | **p** |
| Age, years, mean ± SD | 39.79 ± 12.88 | 42.77 ± 14.42 | NS | – | – | NS | 38.71 ± 12.78 | 43.45 ± 14.69 | NS |
| Female sex, n (%) | 23 (79.3) | 90 (79.6) | NS | 7 (100.0) | 45 (83.3) | NS | 15 (71.4) | 27 (81.8) | NS |
| Comorbidity, n (%) | 19 (65.5) | 75 (66.4) | NS | 6 (85.7) | 39 (72.2) | NS | 13 (61.9) | 22 (66.7) | NS |
| Family history of CSU, n (%) | 6 (20.7) | 17 (15.0) | NS | — | — | NS | — | — | NS |
| Age at first NSAID reaction, years, mean ± SD | 35.86 ± 13.69 | 36.50 ± 14.61 | NS | – | – | NS | 34.90 ± 13.85 | 38.18 ± 14.09 | NS |
| Age at CSU diagnosis, years, mean ± SD | 34.28 ± 13.24 | 35.60 ± 14.21 | NS | – | – | NS | 33.62 ± 12.73 | 37.76 ± 14.72 | NS |
| Interval between CSU onset and first NSAID reaction, months, median (IQR) | 24 (12–48) | 24 (12–48) | NS | 24 (12–28) | 24 (12–60) | NS | 18 (12–60) | 24 (12–48) | NS |
| Number of NSAID-induced reactions, median (IQR) | – | – | – | 1 (1–2) | 1 (1–2) | NS | 2 (1–3) | 2 (1–2) | NS |
| NSAID reaction before CSU onset, n (%) | 4 (13.8) | 16 (14.2) | NS | 0 (0.0) | 9 (16.7) | NS | 4 (19.0) | 5 (15.2) | NS |
| NSAID reaction after CSU onset, n (%) | 13 (44.8) | 61 (54.0) |  | 5 (71.4) | 31 (57.4) |  | 8 (38.1) | 18 (54.5) |  |
| NSAID reaction simultaneous with CSU onset, n (%) | 12 (41.4) | 36 (31.9) |  | 2 (28.6) | 14 (25.9) |  | 9 (42.9) | 10 (30.3) |  |
| Total IgE, IU/mL, median (IQR) | 78 (50–175) | 118.5 (49–300) | NS | 76 (36–175) | 142 (48–314) | NS | 120 (50–175) | 116 (51–276) | NS |
| Anti-TPO positivity, n (%) | 3 (10.3) | 14 (12.4) | NS | 2 (28.6) | 7 (13.0) | NS | 1 (4.8) | 3 (9.1) | NS |
| ESR, mm/h, median (IQR) | 10 (5–13) | 10 (5–17) | NS | – | – | – | 8 (5–13) | 7 (5–14) | NS |
| Blood eosinophil count, /µL, median (IQR) | 120 (90–250) | 130 (80–200) | NS | 100 (40–220) | 105 (40–200) | NS | 190 (100–250) | 170 (100–290) | NS |

*Data are shown as mean ± SD, median (IQR), or n (%), as appropriate. Timing categories for NSAID hypersensitivity relative to CSU onset were compared jointly. Anti-TPO, anti-thyroid peroxidase antibody; ESR, erythrocyte sedimentation rate; other abbreviations as in Tables 1, 3, and 4.*
