# Supplementary material for: NSAID Tolerance Can Be Restored in NECD Even in the Absence of Complete Remission of Urticaria
Source: Clin Transl Allergy. 2026 Oct 21;16(10):e70216. doi: 10.1002/clt2.70216 (PMC13687787; doi:10.1002/clt2.70216)
Supplement: Supplementary file 3 [file CLT2-16-e70216-s004.docx]

**Supplementary Table S3. Omalizumab treatment variables according to ASA provocation outcome (omalizumab-treated group, n = 61).**

|  | **ASA Provocation Outcomes in the Omalizumab Group** | | |
| --- | --- | --- | --- |
|  | **ASA provocation test positive (n = 7)** | **ASA provocation test negative (n = 54)** | **p** |
| Omalizumab dose at testing 150 mg, n (%) | 0 (0.0) | 2 (3.7) | NS |
| Omalizumab dose at testing 300 mg, n (%) | 7 (100.0) | 51 (94.4) |  |
| Omalizumab dose at testing 600 mg, n (%) | 0 (0.0) | 1 (1.9) |  |
| Age at omalizumab initiation, years, median (IQR) | 40 (32–50) | 37 (29–46) | NS |
| Total duration of omalizumab therapy, months, median (IQR) | 24 (24–60) | 36 (14–60) | NS |
| Concomitant antihistamine use, n (%) | 6 (85.7) | 9 (16.7) | p = 0.001 |
| Regular antihistamine use, n (%) | 2 (33.3) | 1 (11.1) | NS |
| Duration of CSU, months, median (IQR) | 84 (36–96) | 69 (48–120) | NS |
| Month of CSU at which omalizumab was initiated, median (IQR) | 24 (12–72) | 36 (12–84) | NS |

*Dose percentages were calculated within each provocation-outcome subgroup and compared jointly. Other abbreviations as in Tables 1 and 3.*
