# Supplementary material for: NSAID Tolerance Can Be Restored in NECD Even in the Absence of Complete Remission of Urticaria
Source: Clin Transl Allergy. 2026 Oct 21;16(10):e70216. doi: 10.1002/clt2.70216 (PMC13687787; doi:10.1002/clt2.70216)
Supplement: Supplementary file 4 [file CLT2-16-e70216-s005.docx]

**Supplementary Figure S1:** Study Timeline and Data Collection Framework
